# Arthrodesis for post-traumatic ankle osteoarthritis leads to a reduced function of the affected limb with changes in gait and pedobarography: a retrospective cross-sectional study

**DOI:** 10.1186/s13018-026-06842-z

**Published:** 2026-04-16

**Authors:** Alexis Brinkemper, Thomas Rosteius, Charlotte Cibura, Lukas Karla, Thomas A. Schildhauer, Christiane Kruppa

**Affiliations:** Department of General and Trauma Surgery, BG University Hospital Bergmannsheil, Ruhr-University Bochum, Bürkle-de-la-Camp-Platz 1, 44789 Bochum, Germany

**Keywords:** Joint fusion, Biomechanics, Arthrosis, Plantar load distribution

## Abstract

**Background:**

Ankle arthrodesis is an established procedure for end-stage ankle osteoarthritis. Besides the known limited functionality, gait and foot arch changes are accompanying pathologies, which are little investigated. The aim of the present study was to determine the influence of ankle arthrodesis on gait, pedobaropraphy and functional outcome.

**Methods:**

Thirtyfive patients with unilateral ankle joint fusion after post-traumatic osteoarthritis and a mean follow-up of 60.8 months (SD 18.5, range 32–92) were included. Primary outcome were a 3-D gait analysis including pedobarography. American Orthopaedic Foot and Ankle Society (AOFAS) Score, douleur neuropathique (DN4), EQ-5D-3 L, numeric pain rating scale (NRS), Olerud-Molander Ankle Score (OMAS), painDETECT, short form 36 (SF-36), short musculoskeletal function assessment—German version (SMFA-D) and a clinical examination were secondary outcomes.

**Results:**

The AOFAS scores showed predominantly fair or poor results (Rearfoot: 48.6% fair, 34.3% poor; Midfoot 42.9% fair, 25.7% poor), while the SMFA tended to reflect mild dysfunction (bother index 31.7, functional index 27.5). Pain-related questionnaires showed a low incidence of pain (DN4 4.1, NRS 2.2, painDETECT 11.6). Kinematic changes were only marginally observed, while the spatio-temporal parameters showed relevant asymmetries between the fused and healthy side. The pedobarography revealed, in addition to other differences, a reduced maximum contact area on the arthrodesis side compared to the healthy side.

**Conclusions:**

Arthrodesis of the ankle joint results in significant limitation of daily activities with reduced function of the affected limb. The impact of ankle arthrodesis go well beyond the loss of extension and flexion, with changes in spatial and temporal movement, weight bearing profile and contact surface. However, results of pain questionnaires show a good result with regard to the main goal of the arthrodesis, the elimination of pain.

**Trial registration:**

DRKS, DRKS00033495. Registered 14 March 2024, https://www.drks.de/DRKS00033495.

## Background

Approximately 1–6% of the world’s adult population suffers from osteoarthritis of the ankle with over 70% of post-traumatic etiology such as fractures close to the joint [1, 2]. Other possible causes include rheumatoid arthritis or the sequelae of nerve diseases. The consequences of osteoarthritis of the ankle for those affected are physical and mental impairments, and are associated with functional and social limitations as well as reduced quality of life [3, 4]. Despite major advances in ankle arthroplasty, the survival rate for ankle replacement is still lower than for knee and hip replacement [1]. Particularly in posttraumatic conditions of young patients with high functional demands, ankle replacement is not a viable option given the current results and survival rates. Therefore, ankle arthrodesis is still considered the gold standard in the treatment of osteoarthritis of the ankle joint [1, 5]. Even though ankle arthrodesis has been a safe and established procedure for years, potential complications remain. The literature describes the potential occurrence of pseudarthrosis, secondary arthrosis in adjacent joints, limited functionality and a change in gait [6, 7]. Many studies compare arthrodesis and total joint arthroplasty [8–11], while far fewer studies analyzed the specific consequences of joint fusion. Studies addressing this issue describe an altered gait pattern and foot geometry [3, 6, 7, 11]. However, the manifestations vary greatly and are not yet fully understood. The aim of the present study was therefore to determine the consequences of ankle arthrodesis using patient reported outcome measures (PROMs) and instrumented gait and pedobarographic analyses, with a special focus on a possible change in the arch of the foot, the foot geometry and the consecutive shortening of the leg due to the resection of the joint.

## Methods

This retrospective, cross-sectional, single center study was performed in accordance with the Declaration of Helsinki and its later amendments. Ethical permission for this study was obtained from the local ethics committee. Included were all patients aged over 18 years, who were treated in our clinic with unilateral internal or external ankle joint fusion and a minimum follow-up of 24 months. Exclusion criteria were age under 18 years, concomitant injuries of the affected limb and secondary transfer with surgical treatment outside our institution. Patients who met these criteria and consented to participate were invited to undergo gait analysis, pedobarography, PROMs and clinical examination.

### Gait and pedobarographic analysis

A three-dimensional (3D) biomechanical gait analysis and pedobarography were conducted on a 5-meter walkway at a self-selected pace. Participants walked back and forth continuously, completing the track 8–10 times. Gait analysis was performed using the 3D MyoMotion system (myoRESEARCH 3.20, Noraxon U.S.A.). Inertial measurement unit sensors, calibrated and positioned on the pelvis, thighs, shanks, and feet, measured anatomical angles and range of motion (ROM) with a sampling frequency of 100 Hz. The ROM and the minimum and maximum peak angles of the joints during gait were analyzed as well as spatial-temporal parameters. Pedobarography was simultaneously performed using the MyoPressure system (myoRESEARCH 3.20, Noraxon U.S.A.) and a pressure distribution platform (Zebris 1.5 FDM, Zebris Medical, Germany) equipped with 11,264 sensors on a 1440 × 560 mm sensor area (resolution: 1.4 sensors/cm^2^) at 100 Hz. To ensure natural gait, the pressure platform was embedded within a wooden walkway. Force data were normalized to body weight and analyzed for the whole foot and with a preconfigured geometric division across ten foot zones: heel medial (HM), heel central (HC), heel lateral (HL), midfoot (MF), metatarsal 1 (M1), metatarsal 2 (M2), metatarsal 3 (M3), metatarsal 4 (M4), metatarsal 5 (M5) and toes (T). All participants walked without assistive devices, and measurements were taken barefoot. All recorded data were averaged for each participant.

### PROMs

To determine the subjective and functional outcomes validated questionnaires (American Orthopaedic Foot and Ankle Society (AOFAS) Score, douleur neuropathique (DN4), EQ-5D-3 L, numeric pain rating scale (NRS), Olerud-Molander Ankle Score (OMAS), painDETECT, short form 36 (SF-36), short musculoskeletal function assessment—German version (SMFA-D)) were completed. Although the AOFAS has not been validated in all contexts, we consider its inclusion justified given its widespread use in the foot and ankle literature.

### Statistical analysis

Descriptive statistics were completed including percent, mean, range and standard deviation (SD). Comparisons between the injured side and contralateral side were performed using the Kolmogorov-Smirnov test, followed by a paired t-test. A Pearson correlation was carried out to examine possible correlations between PROMs and selected gait parameters. All the statistical analyses were carried out using SPSS Statistics 30.0.0.0 (IBM Corp., Armonk, NY: IBM Corp).* P* values ≤ 0.05 were determined as statistically significant.

## Results

### Patients

The study group consisted of 23 (65.7%) males and 12 (34.3%) females with an average age of 57.9 years (SD 11.1, range 32–82). The mean follow-up time was 60.8 months (SD 18.5, range 32–92). 30 patients had a single tibiotalar arthrodesis and five patients had a combined tibiotalar and talonavicular/subtalar arthrodesis. All 35 (100%) of the arthrodesis were caused by post-traumatic osteoarthritis. 27 (77.1%) patients had a chronic course and in eight (22.9%) patients the arthrodesis resulted from acute trauma. Preoperative malalignment in the form of varus or valgus was observed in 12 patients (34.3%), and 12 patients (34.3%) also had a soft tissue compromise due to, for example, open fractures. The clinical scores were available for all 35 patients. 32 patients took part in the gait analysis and pedobarography. In one patient, the joint angle data was compromised, which is why data from only 31 patients was used for the kinematic analysis. Pedobarography data was available for all 32 patients. Patient and injury-related data are shown in Table 1.

**Table 1:** Patients demographics, *n* = 35

	*N* (%) or mean (± SD; range) or median (IQR)
Male	23 (65.7)
Female	12 (34.3)
Age (years)	57.9 (11.1; 32–82)
Follow-up (months)	60.8 (18.5; 32–92)
Height (cm)	176.4 (9.2; 152–193)
Weight (kg)	92.6 (19.9; 57–155)
BMI (kg/m^2^)	29.6 (4.9; 19.3–43.9)
*Comorbidities*	
Smokers	7 (20.0)
Arterial hypertension	14 (40.0)
Diabetes mellitus	8 (22.9)
Asthma	2 (5.7)
Overweight (BMI ≥ 25)	18 (51.4)
Obesity (BMI ≥ 30)	13 (37.1)
Coronary heart disease	3 (8.6)
Polyneuropathy	4 (11.4)
Right side	16 (45.7)
Left side	19 (54.3)
Single tibiotalar arthrodesis	30 (85.7)
Tibiotalar and talonavicular/subtalar	5 (14.3)
Post-traumatic osteoarthritis	35 (100)
Chronic course	27 (77.1)
Soft tissue compromise	12 (34.3)
Preoperative valgus alignment	4 (11.4)
Preoperative varus alignment	8 (22.9)
Preoperative Kellgren Lawrence score	3 (1–4)
Difference in leg circumference 15 cm distal to the knee (cm)	2.9 (2.3; 0–10)
Leg length difference (LLD) (cm)	2.9 (1.9; 0–9)

### PROMs

The AOFAS scores showed predominantly fair or poor results, while the SMFA tended to reflect mild dysfunction. The pain-related questionnaires (NRS, DN4, painDETECT) showed a low incidence of pain. The OMAS to assess the symptoms and function of a patient after an ankle fracture showed tolerable results on average. The SF-36 showed below-average values for the Physical Component Summary (PCS) and ordinary results for the Mental Component Summary (MCS). In the EQ-5D-3 L, the majority of patients reported some problems in the categories of mobility, usual activities, as well as pain and discomfort, while no problems in the categories of self-care and anxiety and depression. Detailed PROMs results are shown in Tables 2 and 3.

**Table 2:** PROMs results, *n* = 35

	*N* (%) or mean (± SD; range)
AOFAS Rearfoot	59.7 (16.1; 19–90)
Excellent	0 (0)
Good	6 (17.1)
Fair	17 (48.6)
Poor	12 (34.3)
AOFAS Midfoot	60.7 (17.4; 19–87)
Excellent	0 (0)
Good	11 (31.4)
Fair	15 (42.9)
Poor	9 (25.7)
DN4	4.1 (2.4; 0–10)
NRS	2.2 (2.3; 0–9)
painDETECT	11.6 (8.8; 0–33)
OMAS	44.7 (18.9; 5–80)
SMFA Bother index	31.7 (23.6; 0-87.5)
SMFA Function index	27.5 (18.0; 1.6–65.5)
SF-36 PCS	37.9 (10.5; 14.2–59)
SF-36 MCS	51.4 (12.7; 24.3–67.0)

**Table 3:** EQ-5D-3 L results, *n* = 35

	Mobility *N* (%)	Self-care *N* (%)	Usual activities *N* (%)	Pain/discomfort *N* (%)	Anxiety/depression *N* (%)
Level 1 (no problem)	7 (20)	22 (62.9)	14 (40)	9 (25.7)	19 (54.3)
Level 2 (some problems)	28 (80)	11 (31.4)	21 (60)	25 (71.4)	15 (42.9)
Level 3 (extreme problems)	0 (0)	2 (5.7)	0 ()	1 (2.9)	1 (2.9)

### Gait analysis

Gait analysis revealed a significantly shortened stance phase (*p* = 0.000), loading response phase (*p* = 0.038), single-limb-support phase (*p* = 0.000) and prolonged swing phase (*p* = 0.042) of the fused side compared to the contralateral side. Step time of the arthrodesis side was also significantly longer (*p* = 0.000). In relation to the entire gait cycle, there was a reduced maximum abduction in the hip on the fused side compared to the opposite side (*p* = 0.039) as well as an increased maximum knee extension (*p* = 0.010). All spatial-temporal and kinematic data are shown in Table 4.

**Table 4:** Spatial-temporal and kinematic analysis, *n* = 31

		Arthrodesis side mean (± SD; range)	Contralateral side mean (± SD; range)	t-test, *p*-value
Stance phase (%)		64.4 (3.4; 56.3–71.0)	69.9 (3.3; 65.1–76.4)	0.000*
Loading response (%)		16.5 (2.5; 11.7–24.2)	17.8 (3.7; 12.9–28.8)	0.038*
Single-limb-support (%)		30.1 (3.2; 23.7–35.0)	35.7 (3.4; 29.2–43.4)	0.000*
Pre-swing (%)		17.8 (3.7; 12.8–28.7)	16.5 (2.5; 11.7–24.6)	0.042*
Swing phase (%)		35.6 (3.4; 29.0-43.7)	30.1 (3.3; 23.6–34.9)	0.000*
Foot rotation (°)		11.2 (7.3; -4.7-28.6)	10.7 (6.4; 0.2–24.2)	0.718
Step length (cm)		44.0 (7.9; 24.7–55.5)	44.0 (9.7; 21.9–59.1)	0.977
Step time (ms)		726.7 (100.1; 553.9-1011.7)	633.2 (79.9; 485.9-895.4)	0.000*
Hip flexion (°)	Max	18.5 (8.1; 2.7–31.1)	17.8 (7.1; 3.8–32.3)	0.593
	Min	−15.4 (8.5; −42.3–1.0)	−17.6 (8.3; −36.6–3.2)	0.227
	ROM	34.4 (7.5; 20.2–48.1)	35.4 (8.4; 16.9–51.2)	0.623
Hip abduction (°)	Max	4.8 (5.1; -5.4-18.4)	7.6 (3.9; 0.5–17.4)	0.039*
	Min	−6.3 (4.5; −14.6–4.2)	−4.8 (3.4; −15.7–0.3)	0.228
	ROM	11.1 (3.8; 4.2–21.1)	12.4 (4.2; 5.1–23.5)	0.174
Hip rotation (°)	Max	4.7 (2.3; -1.1-9.9)	4.3 (1.9; 0.8–9.4)	0.335
	Min	−4.9 (2.4; −10.1–0.3)	−5.2 (1.9; −9.8–1.4)	0.392
	ROM	9.6 (3.5; 3.7–16.3)	9.5 (2.9; 2.9–16.5)	0.880
Knee flexion (°)	Max	48.6 (11.4; 22.8–70.9)	50.9 (8.6; 36.0-67.1)	0.161
	Min	0.4 (4.1; −7.7–7.4)	2.9 (6.3; −6.7–19.3)	0.010*
	ROM	49.0 (11.1; 28.4–70.1)	48.0 (9.6; 30.9–71.3)	0.571
Knee abduction (°)	Max	4.1 (4.5; −6.3–13.5)	4.3 (6.1; −3.7–24.6)	0.873
	Min	−5.6 (4.8; −17.3–2.6)	−6.9 (4.6; −20.8–2.8)	0.327
	ROM	9.7 (4.7; 3.6–20.4)	11.2 (5.9; 3.1–30.9)	0.313
Knee rotation(°)	Max	6.7 (2.9; 2.0-12.5)	7.5 (2.4; 1.3–12.3)	0.124
	Min	−7.5 (2.9; −14.0–3.9)	−6.8 (3.1; −14.1–2.3)	0.392
	ROM	14.2 (4.9; 7.3–22.5)	14.3 (4.2; 4.8–22.9)	0.896

*statistical significant

### Pedobarographic analysis

**Fig. 1:**
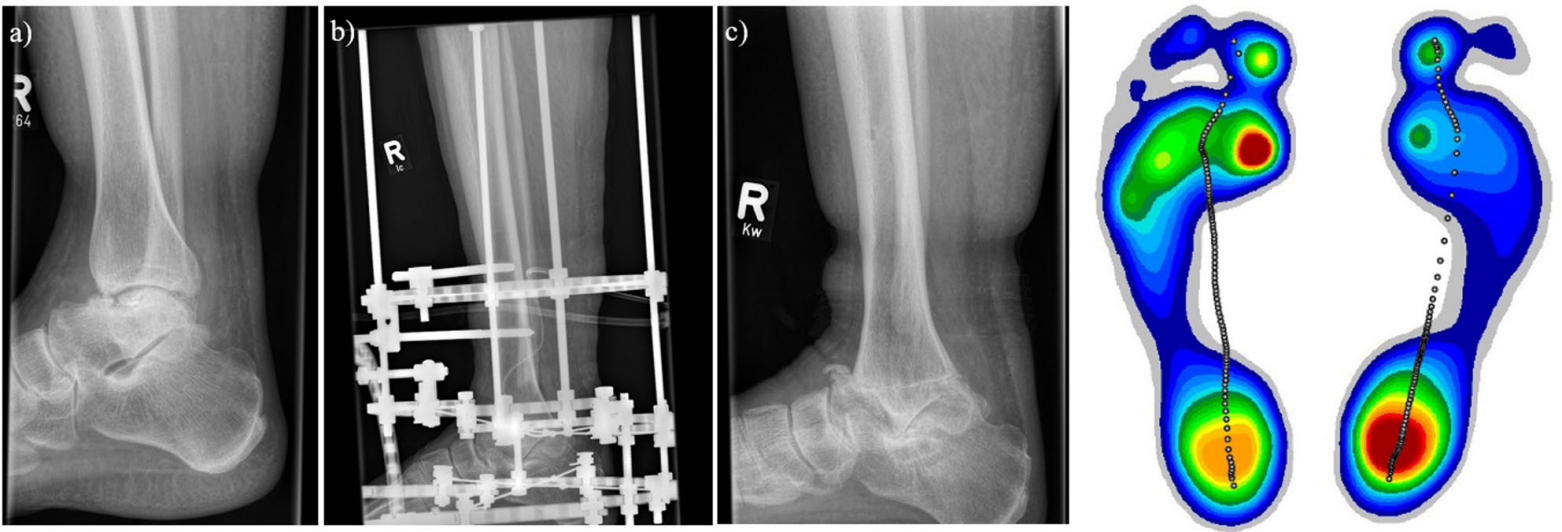
Left side: **a** Patient after talus fracture with talus necrosis and post-traumatic osteoarthritis of the right foot. **b** Right foot fused with external arthrodesis. **c** Right foot 16 months after fusion. Right side: Foot pressure of the same patient 39 months after arthrodesis. Increased pressure on the heel of the fused right side and under M1 on the opposite side

Evaluation of the pedobarography showed an increased maximum force normalized to the body weight on the fusion side for the lateral heel (*p* = 0.039). The contralateral side showed an increased force-time integral for M1 (*p* = 0.010). Overall, there was a reduced mean force (*p* = 0.000) and an increased maximum pressure (*p* = 0.013) in relation to the entire foot on the arthrodesis side. The maximum contact area of the fused foot was reduced compared to the contralateral side (*p* = 0.042). Detailed pedobarography results are shown in Table 5 and an example progression including foot pressure distribution is shown in Fig. 1.

**Table 5:** Pedobarography analysis, *n* = 32

	Arthrodesis side mean (± SD; range)	Contralateral side mean (± SD; range)	t-test, *p*-value
Peak force (*N*/kg)	0.9 (0.1; 0.8–1.1)	1.0(0.1; 0.8–1.1)	0.093
Mean force (N/kg)	0.4 (0.0; 0.4–0.5)	0.5(0.0; 0.4–0.6)	0.000*
Max. pressure(N/cm^2^)	45.9 (17.2; 21.6–91.2)	37.8(12.0; 24.5–65.0)	0.013*
Mean pressure (N/cm^2^)	18.6 (6.1; 10.6–35.1)	17.7(4.5; 9.5–29.2)	0.348
Max. contact area (cm^2^)	129.1 (25.1; 73–197)	136.0(22.3; 102–214)	0.042*
*Peak force (N/kg)*			
Heel medial	0.25 (0.06; 0.13–0.5)	0.25(0.05; 0.14–0.34)	0.944
Heel central	0.13 (0.03; 0.02–0.17)	0.12(0.02; 0.06–0.16)	0.555
Heel lateral	0.23 (0.06; 0.10–0.37)	0.21(0.05; 0.09–0.32)	0.039*
Midfoot	0.19 (0.11; 0.01–0.40)	0.17(0.10; 0.09–0.39)	0.110
Metatarsal 1	0.17 (0.06; 0.01–0.29)	0.19(0.06; 0.08–0.31)	0.104
Metatarsal 2	0.21 (0.08; 0.04–0.40)	0.21(0.06; 0.10–0.32)	0.587
Metatarsal 3	0.11 (0.04; 0.03–0.19)	0.10(0.03; 0.01–0.17)	0.123
Metatarsal 4	0.09 (0.03; 0.03–0.17)	0.08(0.02; 0.04–0.13)	0.090
Metatarsal 5	0.07 (0.03; 0.02–0.14)	0.06(0.03; 0.02–0.14)	0.133
Toes	0.12 (0.06; 0.04–0.29)	0.14(0.06; 0.05–0.28)	0.144
Impulse (%*s)			
Heel medial	10.0 (4.5; 2.3–22.7)	11.1(3.5; 5.3–23.0)	0.189
Heel central	5.3 (2.2; 1.2–11.3)	5.5(1.6; 2.9–9.8)	0.570
Heel lateral	9.0 (3.7; 1.9–18.5)	9.1(2.4; 5.9–15.9)	0.734
Midfoot	7.2 (5.2; 0.5–22.9)	8.0(5.3; 0.9–23.5)	0.323
Metatarsal 1	6.0 (2.8; 0.1–14.0)	7.8(3.0; 2.3–14.9)	0.010*
Metatarsal 2	7.9 (3.7; 0.7–16.8)	8.9(2.7; 2.9–15.3)	0.095
Metatarsal 3	4.2 (1.8; 1.3-8.0)	4.5(1.3; 1.9–7.8)	0.455
Metatarsal 4	3.6 (1.3; 0.8–7.1)	3.6(1.0; 1.7–6.3)	0.930
Metatarsal 5	2.8 (1.1; 0.5–5.1)	2.8(1.0; 1.0-4.9)	0.918
Toes	3.8 (2.1; 0.8–8.2)	4.7(2.4; 1.0-9.5)	0.059

*statistical significant

### Correlation

**Fig. 2:**
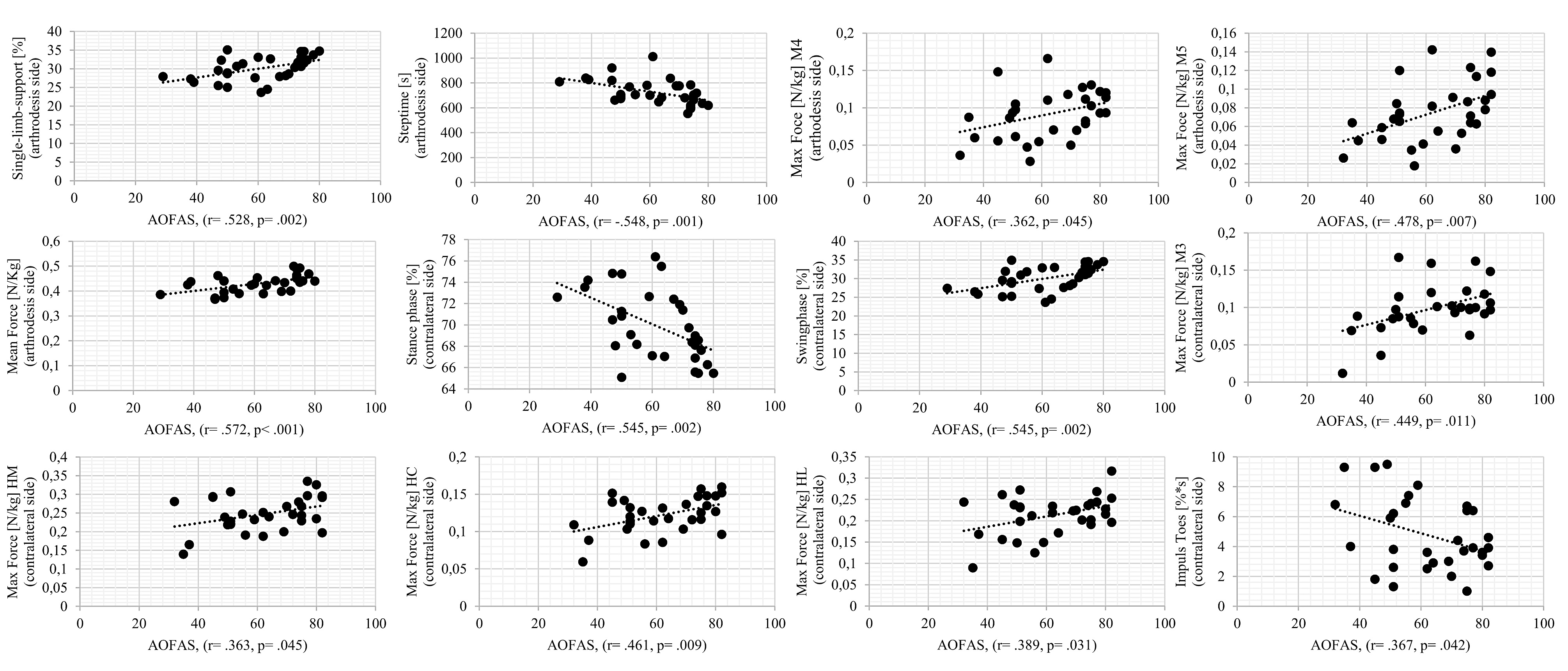
Correlation of AOFAS score and gait analysis and pedobarography data

A correlation analysis between the PROMs obtained and data from the gait analysis and pedobarography revealed relevant results only for the AOFAS score. Patients with a higher AOFAS score, had a longer single-limb-support phase (*r* = 0.528, *p* = 0.002) and a shorter step time (*r* = 0.548, *p* = 0.001) on the fused side. The contralateral side showed a shorter stance phase (*r* = 0.545, *p* = 0.002) and a correspondingly longer swing phase(*r* = 0.528, *p* = 0.002) as the AOFAS score increased. For pedobarography, the AOFAS score was positively correlated with an increased mean force (*r* = 0.572, *p* = 0.001) in the entire foot and increased maximum force below M4 (*r* = 0.362, *p* = 0.045) and M5(*r* = 0.478, *p* = 0.007) on the arthrodesis side. For the contralateral side, there were higher maximum force values for the entire heel (HM, HC, HL) and for M3 (*r* = 0.449, *p* = 0.011) with an increasing AOFAS score. The impulse under the toes on the non-injured side, on the other hand, was lower with an increasing score (*r* = 0.367, *p* = 0.042). Illustrations of the correlations determined as well as the corresponding correlation coefficients and* p*-values are shown in Fig. 2.

## Discussion

Ankle arthrodesis is a safe and established procedure for end-stage ankle osteoarthritis. However, limited functionality and a change in gait and adjacent joints are potential consequences [5]. The aim of the present study was to determine the consequences of ankle arthrodesis using instrumented gait and pedobarographic analyses as well as PROMs.

Our results show satisfactory results after ankle fusion in terms of pain reduction after an average follow-up of five years. However, when considering functionality scores, our collective shows lower values than most other reports: for example, Hendrickx et al. reported AOFAS rearfoot scores of 67 [5]. Schuh et al. also presents scores of 79 and Akra and Adedapo even of 84 [3, 12]. One possible reason for the better results could be that these studies only included patients with isolated tibiotalar arthrodesis, whereas our collective also included a small number of patients with combined tibiotalar and talonavicular/subtalar arthrodesis. Furthermore, 100% of our group consisted of post-traumatic arthrodesis, whereas this proportion was 85% [5], 87% [3] and 0% in the case of Akra and Adedapo [12]. In addition, in the case of Akra and Adedapo [12], only nine patients were included, which appears to be of little conclusive value. In contrast, Fuentes-Sanz et al. showed even worse results than our participants, with an AOFAS score of 50 [7]. In this study, as in ours, the entire collective consisted of post-traumatic arthrodesis. In addition to post-traumatic genesis, nearly a third of our sample population exhibited preoperative malalignment, which may indicate a more complex patient population with lower functional scores.

In the SF-36 our participants showed a PCS of only 37.9 while Hendrickx et al. and Akra and Adedapo showed 43 and 49, respectively [5, 12]. Interestingly, the physical impairments are in contrast to the mental component of the SF-36 where our participants showed normal scores. This indicates that, despite the residual impairment, quality of life after arthrodesis improved compared with the previous condition, probably due to the improvement in pain symptoms. Hendrickx et al. also found the same phenomenon in their study [5].

The fusion of the ankle joint is logically accompanied by a change in the biomechanics of the foot and resulting adaptation mechanisms when walking. In our collective, kinematic changes were only marginally observed, while the spatio-temporal parameters showed relevant asymmetries between the fused and healthy side. In agreement with Zygogiannis et al. [13] and Chopra et al. [11] we found a reduced stance phase on the arthrodesis side compared to the healthy side as well as a shortened loading response phase and single-limb-support phase. In contrast, Wu et al. and Fuentes-Sanz et al. reported a longer stance phase and single-limb-support phase on the operated side [6, 7]. A reduced stance phase is also known in patients with LLD [14], and our patients showed a mean LLD of 2.9 cm. It should be noted that most of our participants wear orthopaedic shoes with height compensation in everyday life, but this was not the case in the current measurement. We suspect that the reduced stance phase is a mechanism to keep painful strain as short as possible. This is supported by the reduced contact area of the entire foot on the arthrodesis side that was revealed in pedobarography. Horisberger et al. [15] also saw a reduced contact area in their study of patients with end-stage ankle osteoarthritis and interpreted it as avoidance of weight bearing and therefore pain. Like us, Fuentes-Sanz et al. [7] found this to be the case in the fused foot for the heel, midfoot and forefoot, even though in their study this was not statistically significant. This might be a kind of trained gait that is difficult to abandon even after surgical treatment because the subconscious fear of pain persists. Another interpretation of the reduced contact area lies in the alignment of the ankle. As already mentioned, some of our patients had a preoperative malalignment. An incompletely corrected preoperative varus or valgus, which is compensated for in everyday life by orthopaedic shoes and insoles, could be one reason for the altered foot contact.

One consequence of the smaller contact area is the increased maximum pressure, which was also shown in pedobarography. We further reveled an increased maximum force for the lateral heel on the arthrodesis side. A relative lateral displacement, in this case of the maximum pressure, on the operated side in the arthrodesis group was also observed by Chopra et al. [11]. This supports the theory of varus position resulting in a lateral force shift. The contralateral side in our collective in turn has an increased force-time integral under M1 due to the relative longer stance time compared to the fused side, which is also an argument in favor of an incorrect lateral loading of the operated side. The mean force on the healthy side is also higher than on the fused side. As mentioned before, differences in gait and therefore foot loading is known from people with LLD. Perttunen et al. stated that the loading of the long limb is greater, which is in line with our results [14]. In their study of patients with end-stage osteoarthritis of the ankle, Horisberger et al. cite atrophy of the muscles surrounding the ankle joint as a further explanation for the change in plantar pressure, and that these patients exhibit considerable muscle weakness [15]. In our clinical survey, a leg circumference difference of 2.9 cm was found 15 cm distal to the inner knee joint gap, which supports this assumption. Other studies comparing fused and unfused foot in contrast to our findings and Chopra et al. [11] did not find differences in kinetic parameters [3, 7]. Different examination methods and evaluation models with regard to foot zone categorization may be one reason for the different results. Furthermore, in one case no normalization to body weight was carried out, which makes comparability more difficult.

Our results show differences in the gait pattern between the fused and contralateral side. The correlations with the AOFAS score also show a direct connection to the general condition after arthrodesis. A better AOFAS score was associated with a longer single-leg stance phase and less step time on the arthrodesis side as well as with higher mean force and maximum force under M4 and M5. This shows that the better the result of the arthrodesis, the more the patients are able to put weight back on the affected foot.

One limitation of the study is its retrospective design, another is the partial accompanying arthrodesis, and, in our opinion, a complex population with post-traumatic genesis, preoperative malalignments, soft tissue compromise, as well as many comorbidities and high body weight, which may not be generally applicable. Further limitations are the number of participants, that three patients did not take part in the gait analysis and pedobarography and the lack of a control group of healthy individuals of the same age and sex to compare gait parameters on the contralateral side, since this side serves as an internal control but may not be representative of a completely healthy population.

## Conclusion

Arthrodesis of the ankle joint results in significant limitation of daily activities with reduced function of the affected limb, according to the functional scores. The gait pattern showed significant differences between the fused side and the contralateral side. Thus, the impact of ankle arthrodesis clearly goes beyond the loss of extension and flexion, which should be mentioned to the patient preoperatively. However, the results of the pain outcome measurements show a satisfactory reduction of generalized and load-dependent pain, which is undoubtedly the main target of ankle arthrodesis.

## Data Availability

All data supporting the findings of this study are available within the paper and its Supplementary Information.

## Abbreviations

PROMs: Patient reported outcome measures
3D: Three-dimensional
HM: Heel medial
HC: Heel central
HL: Heel lateral
MF: Midfoot
M1: Metatarsal 1
M2: Metatarsal 2
M3: Metatarsal 3
M4: Metatarsal 4
M5: Metatarsal 5
T: Toes
AOFAS: American Orthopedic Foot and Ankle Society
DN4: Douleur neuropathique
NRS: Numeric pain rating scale
SF36: Short form 36
SMFA-D: Short musculoskeletal function assessment
SD: Standard deviation
LLD: Leg length difference
